# Alumina Ceramic Nanofibers: An Overview of the Spinning Gel Preparation, Manufacturing Process, and Application

**DOI:** 10.3390/gels9080599

**Published:** 2023-07-25

**Authors:** Meng Xia, Shuyu Ji, Yijun Fu, Jiamu Dai, Junxiong Zhang, Xiaomin Ma, Rong Liu

**Affiliations:** 1 School of Textile & Clothing, National & Local Joint Engineering Research Center of Technical Fiber Composites for Safety and Health, Nantong University, Nantong 226019, China; mengxia0510@126.com (M.X.); jishuyu2015110111@163.com (S.J.); fuyj@ntu.edu.cn (Y.F.); jmdai@ntu.edu.cn (J.D.); 2 National Equipment New Material & Technology (Jiangsu) Co., Ltd., Suzhou 215100, China; 13914089968@163.com

**Keywords:** alumina nanofibers, alumina spinning gel, electrospinning, solution blow spinning, centrifugal spinning, application areas

## Abstract

As an important inorganic material, alumina ceramic nanofibers have attracted more and more attention because of their excellent thermal stability, high melting point, low thermal conductivity, and good chemical stability. In this paper, the preparation conditions for alumina spinning gel, such as the experimental raw materials, spin finish aid, aging time, and so on, are briefly introduced. Then, various methods for preparing the alumina ceramic nanofibers are described, such as electrospinning, solution blow spinning, centrifugal spinning, and some other preparation processes. In addition, the application of alumina ceramic nanofibers in thermal insulation, high-temperature filtration, catalysis, energy storage, water restoration, sound absorption, bioengineering, and other fields are described. The wide application prospect of alumina ceramic nanofibers highlights its potential as an advanced functional material with various applications. This paper aims to provide readers with valuable insights into the design of alumina ceramic nanofibers and to explore their potential applications, contributing to the advancement of various technologies in the fields of energy, environment, and materials science.

## 1. Introduction

According to the current research status, alumina ceramic nanofibers have attracted widespread attention. Alumina ceramic nanofibers have many excellent properties, such as good flexibility, high specific surface area, and the strong adsorption of nanomaterials; alumina nanofibers also show many excellent properties of high-temperature oxidation resistance, excellent mechanical properties, and outstanding chemical stability. These excellent characteristics make it widely used in the fields of high-temperature heat insulation, separations, sound absorption, catalysis, etc.

Alumina ceramic nanofibers generally refer to fibers with an alumina content of more than 70% [1]; the remaining components usually consist of silica, boron nitride, iron oxide, zirconia, etc. Alumina ceramic nanofibers are polycrystalline inorganic fibers, whose main crystal shape can be α-, γ-, δ-, and θ-alumina [2,3]; among them, α-Al_2_O_3_ is the most thermodynamically stable of the alumina crystal forms, and γ-Al_2_O_3_ is almost completely converted to α-Al_2_O_3_ at high temperatures above 1300 °C [4]. Alumina ceramic nanofibers with different properties can be prepared by several different methods. For example, the Cu-Al_2_O_3_ nanofiber membrane prepared by electrospinning has the advantages of high mechanical strength, good flexibility, high catalytic activity, and high porosity, which can be used for the removal of organic pollutants; another example is the Al_2_O_3_-stabilized nanofibers prepared by solution blow spinning. ZrO_2_ fiber air filter paper has excellent flexibility and foldability. With the appropriate production process or post-treatment method, alumina fiber can also be processed into fiber film, fiber cotton, fiber felt, fiberboard, fiber paper, etc. The fibers can also be processed into different forms, such as two-dimensional long or short ceramic fibers, thin films, three-dimensional ceramic fiber aerogels, etc. These different forms of fibers have different uses; for example, two-dimensional long or short ceramic fibers are often used as structural reinforcements for composite materials and lining materials for high-temperature insulation. Three-dimensional ceramic fiber aerogels are commonly used in the field of sound absorption to reduce the noise pollution caused by traffic and engineering construction. Furthermore, because of their high strength and some special electrical and optical properties, alumina ceramic nanofibers can also be used as functional materials in some special fields [5,6]; for example, alumina ceramic nanofibers can be used as catalyst carriers to prepare catalyst materials, to achieve the catalytic degradation of pollutants in the environment. Alumina fiber can also be used to blend with other polymers to obtain battery separators through a certain process, making batteries safer under high temperature conditions.

**Figure 1:**
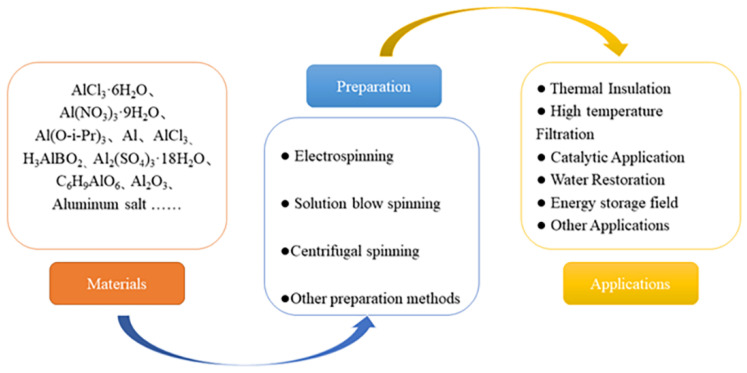
Alumina nanofibers are described in terms of the materials, preparation methods, and applications.

In this paper, the preparation of alumina spinning gel, spinning processes, and their application are reviewed (Figure 1). The material system for preparing alumina spinning gel is firstly introduced, and then the methods for preparing alumina ceramic nanofibers, including centrifugal spinning, electrostatic spinning and solution blowing, are described. Then, the applications of alumina nanofibers in thermal insulation, high temperature filtration, catalysis, water remediation, and energy storage are mainly introduced. Finally, the future development and prospects of alumina ceramic nanofibers are presented, which provides a certain reference value for further research on alumina ceramic nanofibers.

## 2. Preparation of Alumina Spinning Gel

The preparation of alumina spinning gel plays a crucial role in the fabrication of alumina ceramic nanofibers. The selection of the raw materials, their ratios, aging time, and concentrations in the precursor solution can significantly impact the morphology, structure, and properties of the resulting nanofibers during the subsequent spinning process.

In the preparation process of alumina nanofibers, alumina spinning gel is one of the key links. In the preparation of alumina spinning gel, aluminum chloride hexahydrate, aluminum isopropyl oxide, aluminum sulfate, aluminum powder, and aluminum nitrate are generally used as the experimental aluminum sources. During experiments, the alumina spinning gel is prepared by using polyvinyl pyrrolidone (PVP), polyvinyl alcohol (PVA), polyethylene oxide (PEO), polyacrylic acid (PAA), and other polymers as support materials or fiber-forming materials. Then, according to the experimental requirements, researchers add other experimental aids such as acetic acid, hydrochloric acid, ethylene glycol, etc., to prepare an alumina spinning gel that meets the experimental requirements, and then, they move through the preparation process of nanofibers, such as electrospinning, solution jet spinning, centrifugal spinning, etc., to prepare the alumina nanofibers. The ratio of the alumina spinning gel is also adjusted according to the requirements of the synthesis, and according to the shape and structure of the required product, the experimental conditions are also different in the subsequent drying and calcination. Table 1 lists the formation and experimental conditions of the precursor sol for the preparation of the alumina nanofibers.

## 3. Methods for the Preparation of Alumina Nanofibers

Compared with other oxide nanofibers such as silicon dioxide (SiO_2_), zirconia (ZrO_2_), titania (TiO_2_), etc., the research on alumina nanofibers is still in the early stage. The preparation of alumina nanofibers is mainly via electrospinning, solution blowing, and centrifugal spinning. In addition, atomic layer deposition, polymer conversion, and several other methods have been reported for the preparation of alumina nanofibers.

### 3.1. Electrospinning

Electrospinning is a commonly used technique for producing nanofibers with a uniform diameter distribution. It involves the use of a strong electrostatic field to draw out the polymer solution or melt from a needle, forming a cone-shaped droplet known as the “Taylor cone”. From the tip of the Taylor cone, a fiber filament is extended, which then solidifies to form nanofibers [7,8,9]. The diameter, morphology, and structure of the electrospun fibers are influenced by various parameters such as the solution viscosity, the strength of the electrostatic field, the solution extrusion rate, the receiving distance, the temperature, and the humidity of the working environment. These conditions can be optimized to tailor the properties of the resulting nanofibers for a wide range of applications, including tissue engineering, drug delivery, filtration, and sensors. Electrospinning is a widely accepted and effective method for producing nanofibers with unique properties, characterized by their high surface-to-volume ratio and fine nanoscale structure [10,11,12,13,14,15,16,17].

When using electrospinning, nanofibers for different applications can be produced using materials from different sources. For example, Mujiba et al. [18] prepared an SiOC-based PDC fiber pad using three different components of pre-ceramic polymers as raw materials. The fiber pad can be used in supercapacitors and lithium-ion batteries (LIB). When used as a supercapacitor, its maximum specific capacitance is 50 F g^−1^, and the capacity retention rate is 100% after the 2000th cycle; the maximum reversible capacitance when used as a LIB has also been improved. Altinkok et al. [19] used electrospinning process to prepare composite nanofibers using phosphorylated polyvinyl chloride and polyethyleneimine as raw materials. The nanofibers can be used in the biological field. They can provide a safer surface for platelets and have a lower hemolytic activity. Xu et al. [20] used composite citric acid and propionate ligands as template-free prerequisites to obtain mesoporous MgO ceramic fibers by electrospinning. The BET-specific surface area of MgO ceramic fiber can reach 232.4 m^2^/g, the total pore volume of the fiber can reach 0.560 cm^3^/g, the adsorption of Pb (II) and Cd (II) is excellent, and the mobility and separability in solution are excellent, which can reduce energy consumption and save costs.

In the preparation of alumina nanofibers, electrospinning has been utilized. For instance, Dai et al. [21] fabricated fibers composed of boric acid/aluminum oxide PVA using electrospinning, followed by high-temperature calcination to obtain aluminum oxide fibers with a diameter of approximately 550 nm. The combustion temperature significantly influences the structure and morphology of the fibers in this process. Similarly, Kang et al. [22] employed aluminum nitrate, binal alcohol, water, and anhydrous ethanol as raw materials to create an alumina spinning gel with consistent viscosity. The precursor nanofiber was obtained via electrospinning and then subjected to high-temperature burning to obtain nano-diameter alumina fibers. However, electrospinning is only suitable for alumina solutions with relatively low viscosity due to the weak electrostatic field force. Consequently, electrospun alumina fibers may contain less solid alumina, resulting in smaller fiber diameters.

When researchers tried to improve the flexibility of alumina nanofibers, they found two ways to achieve this. One was to add organic compounds, such as PAN, to the alumina solution. The addition of such a substance can improve the continuity of spinning, resulting in more continuous fibers. The second was to add inorganic additives to the solution; the addition of inorganic additives can hinder the growth of the alumina particles in the followup high-temperature calcination process, thus significantly enhancing the flexibility of the fiber. Yu et al. [23] prepared an alumina spinning gel using PAN, DMF, and aluminum 2, 4-glutarate as raw materials. Then, the precursor fiber was prepared by electrospinning, and the precursor fiber was calcined at 1200 °C in a muffle furnace to obtain alumina nanofibers with a fiber diameter of 150–500 nm. The experimental results showed that the continuity of the nanofibers could be affected by the addition of a polymer. Zhang et al. [24] dissolved aluminum chloride hexahydrate and aluminum powder in deionized water, used calcium chloride and silicon dioxide as a two-component additive to synthesize alumina spinning gel, and then obtained alumina nanofibers by electrospinning. The authors showed that the addition of calcium chloride and silica could prevent the phase transition of alumina and control the grain sizes. The alumina nanofibers obtained by calcination at high temperature had a smaller grain size, lower thermal conductivity, and better flexibility than those without two-component additives.

**Figure 2:**
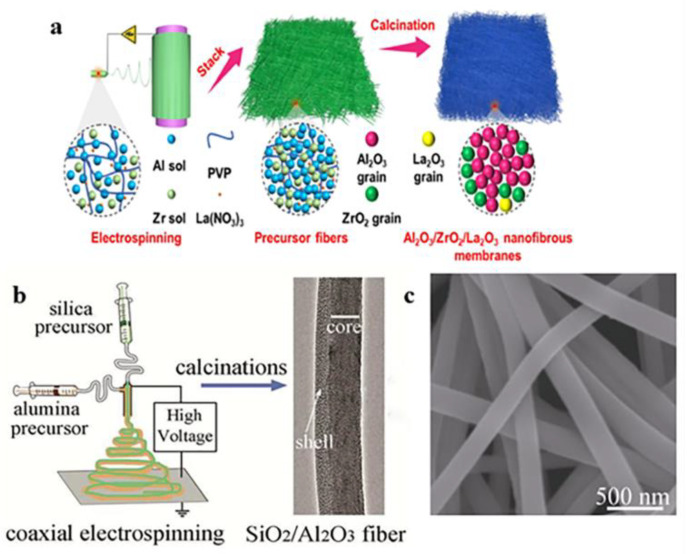
(**a**) Flow chart of the preparation of Al_2_O_3_/ZrO_2_/La_2_O_3_ nanofiber membranes. (**b**) Schematic diagram of a coaxial electrospinning device. (**c**) SEM of silica/mesoporous alumina fibers. (**a**) Reprinted with permission from reference [28]. (**b**,**c**) Reprinted with permission from reference [33].

When the researchers studied the properties of a single alumina nanofiber, they proposed that combining two or more components could obtain composite alumina nanofibers with improved performance. The composite components are different, the nanofibers obtained are different, and their performance is also very different [25,26]. Li et al. [27] prepared NiO/γ-Al_2_O_3_ composite nanofibers with a diameter of 253–391 nm by electrospinning and high-temperature calcination using nickel acetate, aluminum acetate, and PVP materials. This composite fiber can be used in various photocatalytic applications in air/water pollution, and its catalytic efficiency is comparable to that of TiO_2_ particles. Li et al. (Figure 2a) [28] prepared elastically compressible Al_2_O_3_/ZrO_2_/La_2_O_3_ nanofiber membranes by electrospinning and muffle furnace calcination. Al_2_O_3_/ZrO_2_/La_2_O_3_ nanofiber films spun by electrospinning have high elasticity and compressibility at polar temperatures, such as −196 to 1400 °C. In addition, because of their low thermal conductivity and high operating temperature, they can be used as candidate materials in the field of insulating fireproof clothing.

In addition, coaxial electrospinning can also be used in the preparation of composite alumina nanofibers [29,30]. Coaxial electrospinning differs from traditional electrospinning in that coaxial spinnerets have one or more metal needles than traditional spinnerets. The spinning process of coaxial electrospinning is to inject two or three different spinning solutions into different flow channels respectively and then obtain core–shell composite fibers under the action of an electrostatic field [31,32]. Using silica and mesoporous alumina as different components, Wang et al. (Figure 2b,c) [33] prepared a silica/mesoporous alumina core–shell fiber film by coaxial electrostatic spinning. Then, the adsorption capacity of the alumina shell was increased by high-temperature calcination in a muffle furnace. The fiber membrane with core–shell structure can adsorb Congo red well and has high mechanical properties, which is easy to recover and use.

**Figure 3:**
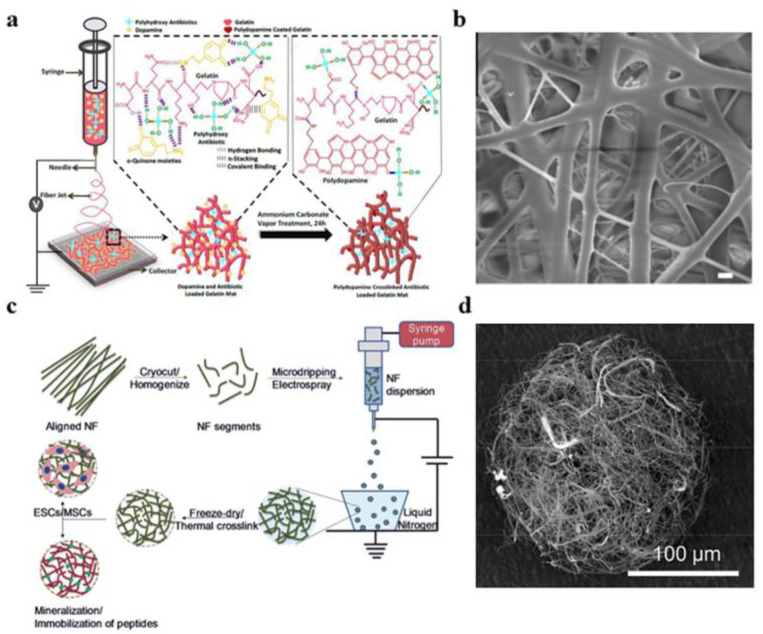
(**a**) Preparation process of nanofibers. (**b**) SEM image of an electrospun gelatin pad. (**c**) Schematic diagram of electrospinning to prepare nanofiber microspheres. (**d**) SEM image of PCL-gelatin nanofiber microspheres (G). (**a**,**b**) Reprinted with permission from reference [36]. (**c**,**d**) Reprinted with permission from reference [43].

In the preparation of alumina nanofibers with uniform diameter distribution, electrospinning is generally used (Figure 3a,b) [34,35,36,37]. However, it is undeniable that this process still has some disadvantages, such as its low speed (less than 1 mL/h), high energy consumption [38,39], and metal needles that are difficult to clean. Therefore, the needleless electrospinning process not only improves the spinning efficiency but also solves the problem of cleaning the metal needles [40]. The principle of needleless electrospinning is to change the transmission mode of the solution during the electrospinning process, so that the spinning liquid is in full contact with the spinning roller; then the spinning roller sprays out nanofibers under the action of the electric field, thereby improving the output of the nanofibers. Wang et al. [38] optimized the needleless electrospinning process on annular spinnerets by the airflow method. The authors examined the effects of the airflow velocity and airflow location on the nanofiber quality and yield. Yu et al. [41] reviewed the advances in needle-free electrospinning, including the structure of the spinners, the spinning efficiency, and the nanofiber quality. They also showed examples of high-throughput devices, as well as industrial devices that could be used in drug delivery, environmental engineering, and more. Jirsak et al. [42] introduced the main research and development directions of needle-free electrospinning and also studied the traditional electrospinning and needle-free electrospinning processes, respectively, explaining the differences between them. Wei et al. (Figure 3c,d) [43] prepared nanofiber membranes by needle-free electrostatic spinning. However, multiple jets were formed at the edge of the spinneret, and the size of the applied electric field affected the number of jets, generally showing a positive correlation. Therefore, needle-free electrostatic spinning can be used to prepare a wide range of nanofiber membranes.

In general, the flexibility of alumina nanofibers prepared by electrospinning is better, but the fiber solid content produced by electrospinning is small, and the spinning speed is low, resulting in a low spinning efficiency. Therefore, the electrospinning process still needs further research in the preparation of alumina nanofibers.

### 3.2. Solution Blow Spinning

**Figure 4:**
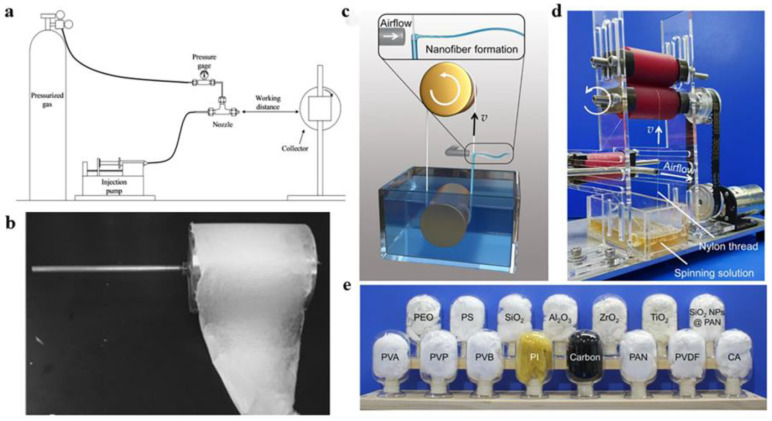
(**a**) Solution blow spinning equipment diagram. (**b**) Non-woven fiber felt prepared by the airflow spinning method. (**c**) Schematic of the needleless KV-SBS device. (**d**) Diagram of spinning for the needle-free KV-SBS equipment. (**e**) Photo of various nanofiber sponges made by KV-SBS. (**a**,**b**) Reprinted with permission from reference [44]. (**c**–**e**) Reprinted with permission from reference [47].

Solution blow spinning, also known as airflow spinning, uses high-speed airflow to prepare continuous nanofibers. The core of the airflow spinning method is that the jet of polymer is stretched by the high-speed airflow, and under the action of the shear force, the polymer jet undergoes a bending and unstable movement; at the same time, the solvent in the alumina spinning gel gradually volatilizes, solidifies, and deposits on the receiving device to form micro-nanofibers (Figure 4a,b) [44,45,46].

In the traditional solution spinning process, the polymer solution is extruded from the tip of the needle and is stretched by gas shear force; then, the solvent evaporates to obtain the fiber. However, Li et al. (Figure 4c–e) [47] developed the needle-free KV-SBS system, which can greatly improve the spinning rate, to realize the large-scale production of nanofibers. It uses a roll-to-roll system to transfer the spinning gel used to make the nanofibers. The spinning system consists of a drum, a spinning liquid container, nylon yarn, and a gas pipe perpendicular to the pipeline, which provides high-speed purging to continuously bring the spinning gel with a certain viscosity out of the container by adhering to the nylon wire on the container. The spinning gel is shaped under the shear force of the high-speed airflow and sprayed rapidly; it is then rapidly stretched to form nanofibers, which are collected on the device. The rapid stretching of the nanofibers is accompanied by the evaporation of solvents. After continuous blowing, the threads are refurbished and returned to the vessel, thus maintaining an uninterrupted flow of the spinning gel.

The difference between the solution-blowing process and the electrospinning method is that airflow spinning uses high-speed airflow, while electrospinning uses electrostatic fields. In addition, in the fiber collection process, the fiber collector of the solution blow spinning is different from the electrospinning when preparing nanofibers, which requires conductive materials as a collection plate and needs to connect voltage, while the solution blow spinning method can be spun on any substrate, and it can even be collected directly on the human hand to form a nanofiber film [48,49].

**Figure 5:**
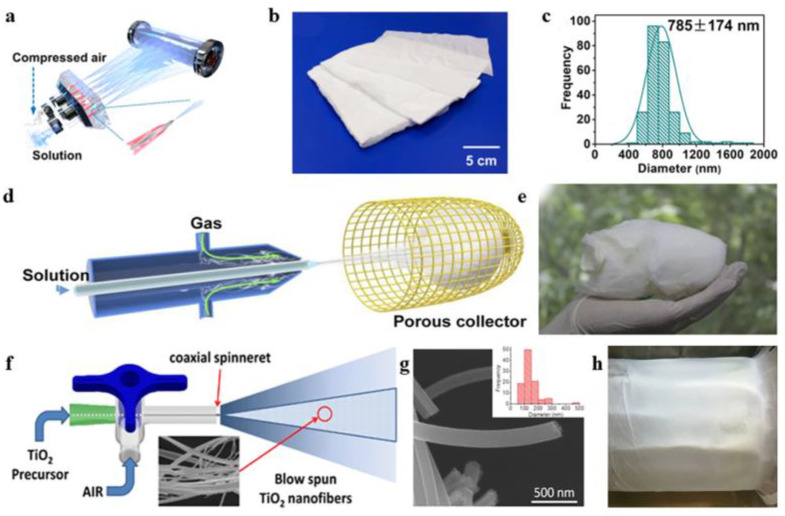
(**a**) Solution blowing to prepare Al_2_O_3_-ZrO_2_ fiber air filter paper. (**b**) Optical image of filter papers of different thicknesses. (**c**) Histogram of fibers. (**d**) Solution blow spinning schematic. (**e**) Photo of a precursor sponge of macro size. (**f**) Flow chart of TiO_2_ nanofiber preparation. (**g**) SEM image of nanofibers as well as histograms. (**h**) Image of a drum collector coated with blown nanofibers on the surface. (**a**–**c**) Reprinted with permission from reference [51]. (**d**,**e**) Reprinted with permission from reference [52]. (**f**–**h**) Reprinted with permission from reference [53].

In the preparation of alumina nanofibers, in addition to the most commonly used electrospinning method, the solution blow spinning method can also use high-speed airflow as a driving force to spin alumina spinning gel into alumina nanofibers using a set of simple concentric nozzles. The fibers are quickly processed and collected into different structures, such as fiber membranes or cotton wool-like structures. Mota et al. [50] demonstrated the preparation of alumina nanofibers by solution blow spinning technology. At 500–1200 °C, the nanofibers were heat treated to transform into γ-Al_2_O_3_ and α-Al_2_O_3_. The experimental results also showed that the solution blow spinning produced alumina nanofibers with high efficiency. Jia et al. (Figure 5a–c) [51] developed an Al_2_O_3_-stabilized ZrO_2_ fiber air filter paper through solution blow spinning and high-temperature calcination. The filter paper had excellent properties, such as good flexibility and high thermal stability. At the same time, the diameter of the fibers in the filter paper was roughly 785 nm, and the distribution was narrow. With the addition of alumina, the filter paper showed excellent flexibility and foldability.

In the current research, there are relatively few examples of preparing alumina nanofibers by airflow spinning, but alumina nanofibers belong to a type of ceramic nanofiber, so the method of preparing other ceramic nanofibers by airflow spinning can almost be applied to the production of alumina nanofibers. Wang et al. (Figure 5d,e) [52] prepared a three-dimensional sponge of nanofibers by solution blow spinning. This nanofiber three-dimensional sponge had the advantages of a light weight, high-temperature resistance, high yield, and good elasticity. Ceramic nanofiber sponges are made of many ceramic nanofibers wound and stacked with densities ranging from 8 to 40 mg/cm^3^. It can provide different functions such as photocatalytic activity and thermal insulation. Manuel et al. (Figure 5f–h) [53] obtained mesoporous titanium dioxide nanofibers by airflow spinning. The test results were characterized by the excellent spatial structure and better cell colonization effect of the polylactic acid scaffold prepared by solution blowing and spinning. Wang et al. [54] prepared and studied oxide ceramic nanofibers such as TiO_2_, ZrO_2_, SnO_2_, and BaTiO_3_ by changing the formulations of different airflow spinning precursor solutions and carried out large-scale preparation and assembly of a series of oxide nanofibers. Oxide nanofiber sponges with elasticity at room temperature and high-temperature conditions have been realized, and the oxide nanofiber materials prepared by air spinning and their multidimensional structures have a wide range of application prospects in the fields of mechanics, catalysis, heat insulation, sensing, filtration, and flexible electronics.

Overall, the spinning efficiency of airflow spinning is higher than that of electrospinning in the preparation of alumina nanofibers. The fiber density obtained by air spinning ranges from 8 to 40 mg/cm^3^, and the mechanical elasticity at room temperature and high temperature is excellent.

### 3.3. Centrifugal Spinning

Centrifugal spinning is a method to produce a uniform diameter and regular arrangement of nanofibers, which combines the advantages of electrospinning with and without needles [38,55,56]. The principle of centrifugal spinning is that the polymer melt or solution containing spinnable polymer enters the high-speed rotating chamber from the extruder at a uniform speed. The centrifugal force promotes the spinning solution to be thrown to the nozzle on the rotary table; then, the solution is ejected through a fine hole, the spinning raw material is gradually elongated, the solvent is rapidly volatilized, and finally, it is collected to form dry nanofibers. The properties of the nanofibers prepared by centrifugal spinning are affected by the components of the spinning solution and spinning process parameters. Zhang et al. [38] reviewed the centrifugal spinning process. It was also compared with other nanofiber preparation methods, such as electrospinning, melt blowing [57], two-component fiber spinning [58,59], phase separation [60], the template method [61,62,63,64], and self-assembly technology [65,66,67,68].

Generally speaking, the preparation of alumina nanofibers with different morphologies or structures by centrifugal spinning is affected by many factors, such as solution viscosity, rotary speed, solution extrusion rate, temperature, and humidity, etc. [69,70]. The influence of different factors on the morphology and structure of alumina nanofibers was investigated [56,71,72,73].

Jancic et al. [74] explored the influence of some process parameters on the diameter of alumina fibers during centrifugal spinning, such as the viscosity of the spinning gel and the disk rotation speed. Alumina fibers with different diameters can be prepared by adjusting the above parameters. The results showed that the diameter of the prepared nanofibers was positively correlated with the spinning gel viscosity and rotating speed. Xu et al. [75] selected aluminum chloride and aluminum powder as aluminum sources and prepared A80 polycrystalline alumina fiber by centrifugal spinning. The effects of the colloid viscosity, colloid flow, colloid temperature, heat treatment heating rate, and sintering temperature on the properties of the fibers were studied. The results showed that suitable centrifugal spinning parameters and heat treatment temperatures can obtain better fiber properties. Li et al. [76] developed an empirical model. They tried to influence the diameter of the nanofibers by controlling the parameters of the centrifugal spinning process, such as the spinning gel viscosity, spinning speed, and nozzle diameter. They verified their empirical model by preparing carboxylated chitosan–polyethylene oxide composite nanofibers. The results showed that the nanofibers with uniform diameter could be prepared by the centrifugal spinning method, and the spinning efficiency was high. Duan et al. [77] explored the motion and force of the spinning gel in a spinneret by establishing a parameter model. This study provided a reference for the preparation of nanofibers and equipment optimization. It can be seen from the above studies that the spinning parameters of centrifugal spinning affect the morphology and structure of the nanofibers. For example, as the viscosity of the spinning gel and the rotational speed of the disc increase, so does the diameter of the fiber.

**Figure 6:**
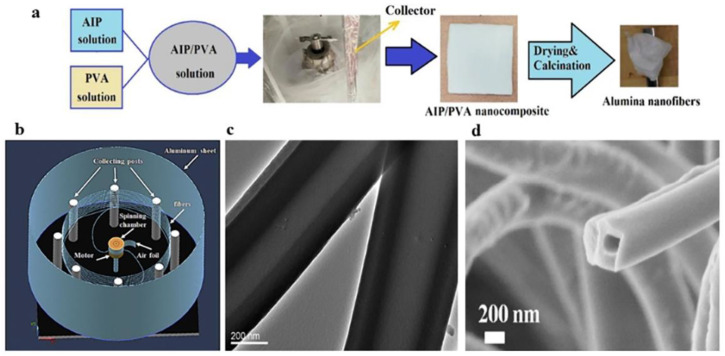
(**a**) Schematic of the alumina fibers’ production process. (**b**) Schematic diagram of centrifugal spinning. (**c**) TEM image of hollow silica nanofibers. (**d**) SEM image of silica nanofibers in hollow section. (**a**) Reprinted with permission from reference [69]. (**b**–**d**) Reprinted with permission from reference [80].

The centrifugal spinning method can prepare alumina nanofibers with excellent performance. Akia et al. (Figure 6a) [69] prepared alumina nanofibers by centrifugal spinning and optimized their properties. In this paper, AIP/PVA composite fibers were prepared by a centrifugal spinning process using aluminum isopropyl alcohol (AIP) and polyvinyl alcohol (PVA) as raw materials, and then alumina nanofibers were obtained by drying and high-temperature calcination. The γ phase and α phase of alumina can be obtained at different calcination temperatures. According to the results of the electron microscopy, the average diameter of the nanofibers calcined at 1200 °C was 272 nm. Centrifugal spinning can also prepare alumina pads for use as high-temperature insulating materials. Sedaghat et al. [70] prepared alumina pads with a nano-microstructure using aluminum chloride hexahydrate and aluminum powder as raw materials by the sol–gel method and the centrifugal spinning process.

As far as the current situation is concerned, there are few experimental examples of alumina nanofibers prepared by centrifugal spinning. However, alumina nanofibers belong to a kind of oxide nanofibers in ceramic nanofibers; so, the process of centrifugal spinning to prepare other ceramic oxide nanofibers is suitable for the production of alumina nanofibers.

Liu used tetra butyl titanate and polyvinylpyrrolidone as raw materials to obtain TiO_2_ nanofibers with a smaller diameter and high photocatalytic performance through centrifugal spinning. During the experiment, TiO_2_ nanofibers with different morphologies were obtained by adjusting different parameters, such as the stirring time of the solution, the spinning gel component, the rotation speed of the disk, etc. TiO_2_ nanofibers with good morphology were obtained by optimizing these parameters [78]. Yanilmaz et al. [79] prepared SiO_2_/PAN films by centrifugal spinning and characterized the membranes of lithium-ion batteries by using different electrochemical technologies. The results showed that the SiO_2_/PAN films were porous and had high wettability and ionic conductivity. Ren et al. (Figure 6b–d) [80] quickly and efficiently prepared multi-layered silica nanofibers by centrifugal jet spinning, which was 500 times faster than electrospinning. Due to the phase separation induced by non-solvent evaporation in the spinning solution, hollow or porous silica nanofibers were formed. The technique can be used to prepare various ceramic materials with multilayer fiber structures on a large scale. Wu et al. [81] selected titanium tetrabutol, ethyl silicate, and polyethylpyrrolidone as raw materials, added propyl trimethoxy-silane (KH-560), and prepared N-Si doped carbon-embedded TiO_2_ composite fibers through centrifugal spinning and heat treatment. According to the SEM image, the fiber fineness was finer when the content of the PVP was low, and the solvent of ethanol was high. With the change in the PVP concentration from 19 wt% to 4 wt%, the diameter of the nanofibers also changed, from the original 948.3 nm to 737.9 nm.

In conclusion, centrifugal spinning shows great prospects in producing alumina nanofibers in industry. It has the advantages of a high spinning efficiency and a high fiber yield. However, in the process of centrifugal spinning, due to the larger pull of centrifugal force, the fiber drafting is not uniform; so, the continuity of the fiber is poor. Therefore, the preparation of alumina nanofibers by centrifugal spinning needs further research.

### 3.4. Other Preparation Methods

Apart from the three commonly used preparation methods of nanometer alumina fibers, there are also other fabrication processes, such as atomic layer deposition (ALD), supercritical fluid drying, hydrothermal growth, molecular layer deposition, anodizing, flame chemical vapor deposition, mercury-mediated techniques, the “pH swing” method, polymer conversion, etc.

**Figure 7:**
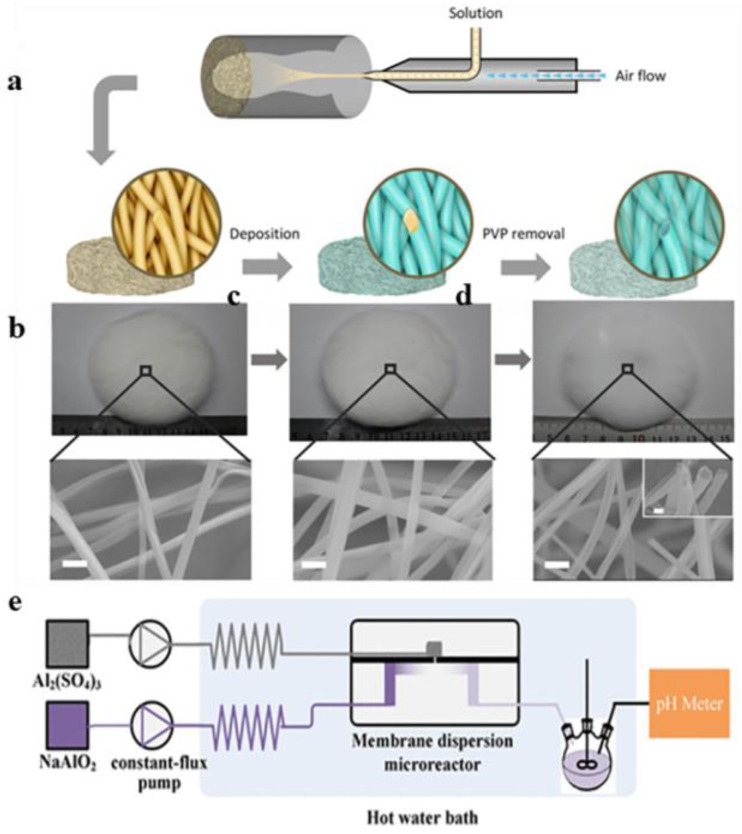
(**a**) Schematic diagram of the Al_2_O_3_ fiber sponge prepared by the ALD method. (**b**) PVP fiber sponge digital image and SEM images. (**c**) Atomic layer deposition of the preparation of the PVP–Al_2_O_3_ composite fiber sponge from a digital image and SEM images. (**d**) Image of the Al_2_O_3_ fiber sponge after template removal. (**e**) Equipment for the preparation of γ-alumina. (**a**–**d**) Reprinted with permission from reference [82]. (**e**) Reprinted with permission from reference [88].

Xu et al. (Figure 7a–d) [82] prepared an Al_2_O_3_ fiber sponge by atomic layer deposition technology (ALD). Firstly, a polyvinylpyrrolidone (PVP) sponge was prepared by the solution blow spinning method; then, ALD was used to deposition a layer of Al_2_O_3_ on the surface of the PVP fiber, and finally, the template was removed by high-temperature calcination to retain the Al_2_O_3_ fiber sponge. The Al_2_O_3_ fiber sponge had high elasticity, low density, and low thermal conductivity. Wang et al. [83] proposed a non-surfactant method to prepare alumina nanofibers, uniformly hydrolyzed aluminum nitrate by hexamethylenetetramine (HTMA), and then synthesized fibrous δ-Al_2_O_3_ by supercritical fluid drying. The results showed that the δ-Al_2_O_3_ nanofibers could be prepared by supercritical fluid drying. They had a diameter of 2 nm and a length of 50 nm. Park et al. [84] used 1-hexadecyl-3-methylimidazolium chloride (C16MimCl) as a templated and co-solvent function for ionic liquids at room temperature to prepare large mesoporous gamma-alumina by heat treatment in an open container without the need to add molecular or organic solvents under ambient pressure. The gamma-alumina nanofibers had a diameter of about 1.5–3 nm and a length of 4060 nm, and the alumina nanofibers had good thermal stability and reasonably acidic sites. Yang et al. [85] synthesized alumina nanofibers in a mercury medium using the mercury-mediated method at room temperature. Through a series of performance tests, it was found that the grown alumina nanofibers were amorphous, with a fiber diameter of 5–15 nm. After 2 h of calcination at 850 °C, the amorphous alumina nanofibers were converted to γ-Al_2_O_3_ nanofibers. Guo et al. [86] used trimethyl aluminum vapor as reactant by flame chemical deposition and formed alumina nanofibers with pure oxygen support in a carbon-containing concurrent diffusion flame. The prepared alumina nanofibers with diameters of 2–10 nm and lengths of 20–210 nm were non-crystalline. In this method, the flame temperature and carbon were very important for the formation of alumina nanofibers, which may affect the concentration of aluminum in the gas phase. Nakajima et al. [87] successfully prepared many high aspect ratio alumina nanofibers by selecting the appropriate electrochemical conditions to control the structural nano characteristics of anodized aluminum nanofibers. Wan et al. (Figure 7e) [88] synthesized fibrous γ-Al_2_O_3_ with large pore volume using NaAlO_2_ and Al_2_(SO_4_)_3_ as reactants by the coprecipitation method. The results showed that the diameter of the nanofibers was about 3.4 nm at the pore volume of 1.52 mL/g. Alumina nanofibers prepared by the coprecipitate method had a narrow pore size distribution and could be used as catalyst support in petroleum refining.

**Figure 8:**
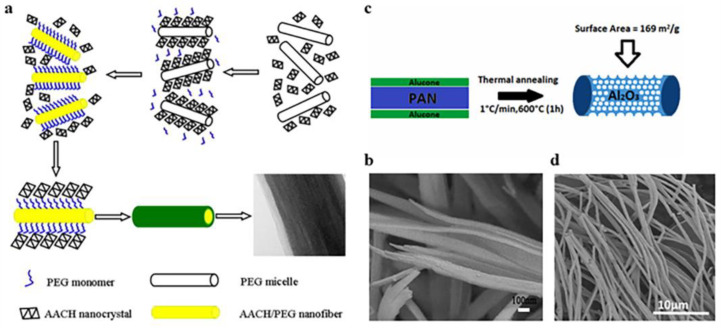
(**a**) Self-assembly mechanism of aluminum bicarbonate amine (AACH)/polyethylene glycol (PEG) microfibers. (**b**) A single broken fiber in the process of calcination in an SEM image. (**c**) The alumina nanofiber preparation process. (**d**) Scanning electron microscopy image of Al_2_O_3_. (**a**,**b**) Reprinted with permission from reference [89]. (**c**,**d**) Reprinted with permission from reference [90].

Zhu et al. (Figure 8a,b) [89] used aluminum nitrate nine-water and urea as raw materials to synthesize AACH precursors through hydrothermal self-assembly and then obtained multilayer Al_2_O_3_ microfibers with a mesoporous structure through thermal decomposition. The average diameter of the alumina microfiber was about 300–500 nm, its specific surface area was large, and its aperture distribution was narrow. Younes et al. (Figure 8c,d) [90] proposed a method of preparing porous alumina nanofibers by electrospinning and molecular layer deposition. Firstly, polyacrylonitrile nanofibers were synthesized by electrospinning, and then aluminum alkyl hybrid films were deposited on the polyacrylonitrile template by molecular layer deposition (MLD). Finally, the template was removed by high-temperature calcination, and porous alumina nanofibers were obtained. The high surface area of the nanomaterial gave it great potential for removing organic dyes. Jose et al. [91] prepared alumina–titanium dioxide nanofibers by pH swing. The method was to change the pH value from 2 to 8, repeat the range several times (e.g., a cycle), add titanium oxysulfate in different ways, and then obtain alumina–titanium dioxide nanofibers by washing, drying, and high-temperature calcination.

## 4. Applications of Alumina Nanofibers

### 4.1. Thermal Insulation

It is well known that alumina ceramic nanofibers have excellent properties of low heat capacity, low thermal conductivity, low creep rate, and high thermal shock resistance, they are especially suitable for thermal insulation and insulation, and the maximum long-term use temperature of fibers with high alumina content can be higher than 1600 °C. So, it is especially suitable for industrial furnaces, aerospace, and other areas that need light insulation materials [92].

**Figure 9:**
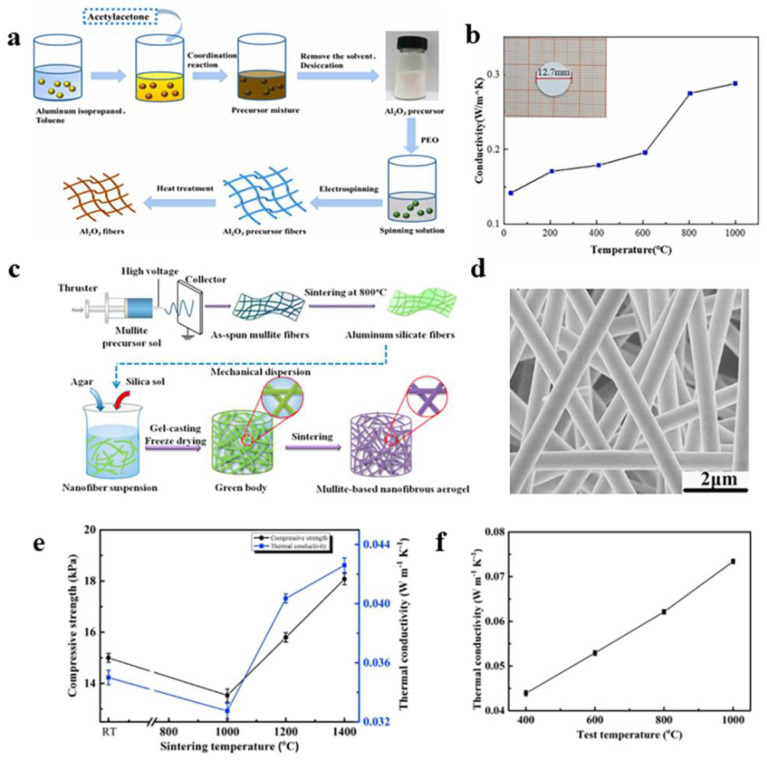
(**a**) Diagram of alumina fiber preparation. (**b**) Thermal conductivity diagram of Al_2_O_3_ fibers. (**c**) Diagram of preparation of Mullite-based nanofiber aerogel. (**d**) SEM of nanofibers before sintering with an alumina/silica molar ratio of 3:1. (**e**) Diagram of the compressive strength and thermal conductivity of the aerogels at different temperatures. (**f**) Thermal conductivity of the mullite-based nanofiber aerogel. (**a**,**b**) Reprinted with permission from reference [93]. (**c**–**f**) Reprinted with permission from reference [97].

Using aluminum isopropyl alcohol and acetylacetone as raw materials, Wang et al. [93] obtained alumina nanofibers with a diameter of about 300–400 nm by electrospinning and calcination, as shown in Figure 9a,b. This fiber had a compact structure and no surface defects. The fiber structure changed from amorphous to γ- Al_2_O_3_ at about 700 °C and then completely to α-Al_2_O_3_ at 1000 °C. The fiber can be used in aerospace and high-temperature industries. Li et al. [94] obtained Al_2_O_3_-SiO_2_ composite nanofibers by using electrospinning. According to the performance test, the alumina composite fiber was transformed into Al_2_O_3_-SiO_2_ composite nanofibers with a diameter of about 300 nm under heat treatment at 1300 °C. The prepared composite nanofibers had low thermal conductivity; so, they could be used in a high-temperature insulation field. Jia et al. [95] prepared a SiO_2_-Al_2_O_3_ composite fiber sponge, which had the characteristics of an anisotropic layered structure and low density. Due to the characteristics of the composite fiber sponges, their thermal conductivity in the direction perpendicular to the fiber layer was low, only 0.034 W m^−1^ K^−1^, when the density was 13 mg cm^−3^, and the temperature was 20 °C. As the density of the sponge increased, so did the thermal conductivity. Yamashita et al. [96] chose cotton fiber as the template to prepare hollow Al_2_O_3_ microfibers by the hydrothermal method. Hollow Al_2_O_3_ microfibers do not contain impurity phases, and their chemical and physical properties indicate that they can be used as insulation materials.

Alumina aerogel has many excellent properties, such as a high specific surface area and porosity, light porosity, good thermal stability, and so on. Alumina aerogel is an excellent high-temperature insulation material. Liu et al. (Figure 9c–f) [97] prepared mullite-based nanofiber aerogel by gel casting and freeze-drying. They used electrospun nanofibers with different alumina/silica molar ratios as the substrate and silica sol as the high-temperature adhesive. Because of its special structure, it still had ultra-low density and room temperature thermal conductivity after heat treatment at 1400 °C; so, it can be used as a thermal insulation material in different thermal insulation environments.

### 4.2. High-Temperature Filtration

With the rapid expansion of industry, particulate pollution is becoming more and more serious, which poses a great threat to human health. Therefore, removing particles has become a top priority in various fields. Alumina nanofibers have the characteristics of high thermal stability, excellent mechanical properties, and high-temperature resistance. Therefore, alumina nanofiber films prepared by electrospinning technology can be used in high-temperature filtration.

**Figure 10:**
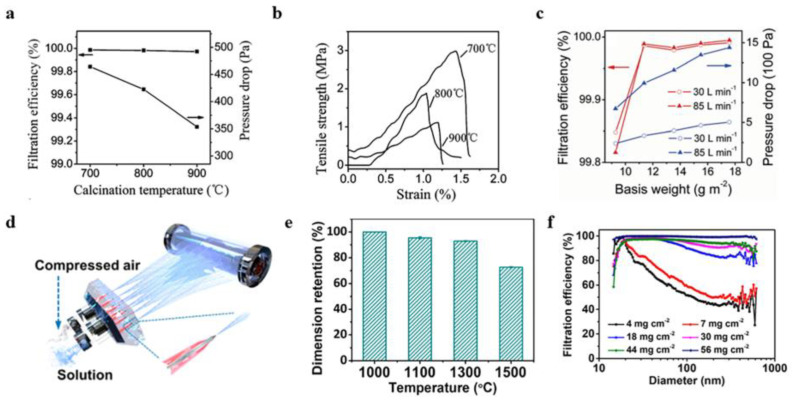
(**a**) Filtration efficiency and pressure drop of the alumina membrane at different calcination temperatures. (**b**) D Tensile stress–strain curves of the γ-alumina fiber films. (**c**) The filtration efficiency and pressure drop vary with the alumina membrane base weight. (**d**) Schematic diagram of the Al_2_O_3_-stabilized ZrO_2_(ASZ). (**e**) Dimensional stability diagram of the ASZ paper. (**f**) Relationship between the filtration efficiency and particle size of the ASZ paper at a 5.4 cm s^−1^ flow velocity. (**a**−**c**) Reprinted with permission from reference [98]. (**d**−**f**) Reprinted with permission from reference [51].

Wang et al. (Figure 10a–c) [98] obtained γ-Al_2_O_3_ nanofiber membranes by electrospinning, in which the fiber diameter was about 230 nm. When the weight of the γ-Al_2_O_3_ nano-fiber membrane was 11.36 g m^−2^, the filtration efficiency of the nano-colloidal particles (300 nm) was very high, up to 99.97%. The results showed that the γ-Al_2_O_3_ nanofiber membranes with high temperature and corrosion resistance could be used as filtration materials in both good and bad environments. Jia et al. (Figure 10d–f) [51] prepared Al_2_O_3_-ZrO_2_ (ASZ) sub-nanofiber air filter paper with good flexibility and high thermal stability by solution wire-blowing and high-temperature calcination. Al_2_O_3_ gave the ASZ fiber a tequad phase and a small particle size; so, the filter paper had excellent flexibility and foldability. Under certain conditions, such as the airflow rate of 5.4 cm^−1^, the filtration efficiency of the ASZ air filter paper for nanoparticles was up to 99.56%. Therefore, this alumina-based air filter material had a large application space in removing particles in high-temperature exhaust gas. Stanishevsky et al. [99] used aluminum nitrate/polyethylene pyrrolidone nanofibers prepared by the free-surface alternating current electrospinning method to prepare alumina nanofibers. Sheets of 100–300 nm thickness were collected at the formation rate of 6.4 g/h of alumina nanofibers. Based on the synthesis of the nano alumina fiber membrane performance testing, they evaluated its potential use in gas filtration.

### 4.3. Catalytic Application

**Figure 11:**
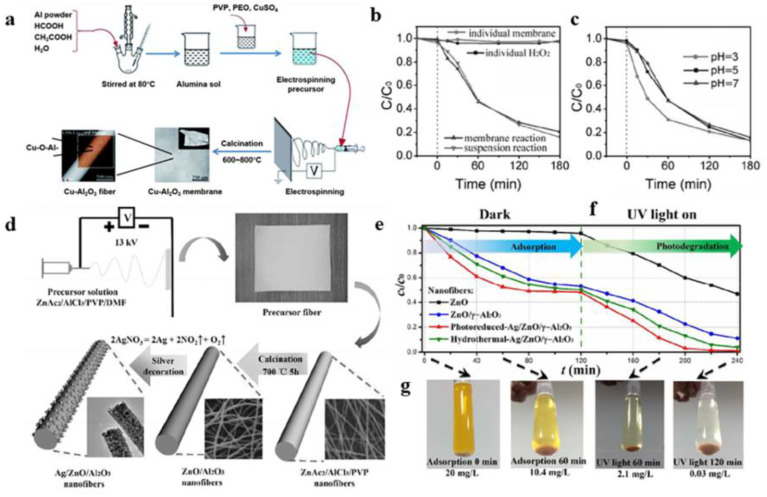
(**a**) Schematic showing the preparation of the Cu-Al_2_O_3_ fibrous membranes. (**b**) BPA removal efficiency with an initial pH of 7.0. (**c**) Effect of pH on the performance of the Cu-Al_2_O_3_-600 membrane. (**d**) Schematic diagram of the preparation of the Ag/ZnO/γ-Al_2_O_3_ nanofibers. (**e**) Schematic diagram of the nanofiber adsorption degradation MO solution. (**f**) Photocatalytic degradation effect diagram. (**g**) Photo of the MO solution prepared by the Ag/ZnO/γ-Al_2_O_3_ nanofibers. (**a**–**c**) Reprinted with permission from reference [104]. (**d**–**g**) Reprinted with permission from reference [105].

As is known, alumina ceramic fiber materials have great application prospects in catalysis due to their large surface area, good chemical stability, and good thermal stability. In general, the application of ceramic fibers in the field of catalysis has two forms. First of all, some materials themselves have good catalytic activity, and when directly processed into fibers, they can be used in the field of catalysis [100,101,102,103]. The second is that ceramic fibers serve as a carrier of catalysts, and catalysts can be added to the ceramic fibers to give the post-processed ceramic fibers catalytic activity (Figure 11) [104,105,106,107]. Alumina ceramic nanofibers are mainly used in the catalytic field in the second form.

When alumina nanofibers are used as a catalyst support to prepare catalyst materials, there are generally two methods. One is to make changes in the process of preparing the alumina spinning gel, adding the catalyst or catalyst carrier directly and then spinning and calcination to obtain nanofibers with catalytic activity. Wang et al. [104] first prepared an alumina spinning gel containing alumina and copper precursors and then prepared a flexible self-supporting Cu-Al_2_O_3_ fiber membrane with catalytic activity by electrospinning. The fiber membrane exhibited high Fenton catalytic activity at a neutral pH. In addition, when tested at a neutral pH containing H_2_O_2_ using a membrane reactor with a 1 wt% Cu-Al_2_O_3_ membrane, the results showed that more than 87% of the BPA degraded within 180 min. The other is to prepare the alumina spinning gel first, then spin it and calcinate it to make the alumina nanofibers as a catalyst carrier, and then load the catalyst onto the nanofibers to obtain nanofibers with catalytic activity. Wu et al. [108] obtained alumina spinning gel by hydrolyzing aluminum trisecondary butanol (ASB) and then prepared alumina nanofibers by improved electrospinning and calcination. According to the electron microscopy, nitrogen adsorption–desorption, and catalysis tests, it was found that the prepared Au/Al_2_O_3_ mesoporous material was an efficient heterogeneous catalyst. Wang et al. [109] also prepared a Pt/Al_2_O_3_ nanofiber membrane catalyst by electrospinning. In this process, the embedding of the Pt nanoparticles and the formation of the Al_2_O_3_ nanofibers were simultaneous. The catalytic tests showed that 100% of the BPA was removed within 60 min when the Pt/Al_2_O_3_ membrane was used for catalysis, and that the CO was fully converted to CO_2_ at 242 °C. Cheng et al. [105] obtained ZnO/γ-Al_2_O_3_ nanofibers by electrospinning and then modified the Ag nanoparticles on the surface to obtain Ag/ZnO/γ-Al_2_O_3_ nanofibers with high photocatalysis. Liu et al. [110] prepared nickel–alumina nanofiber catalysts by electrospinning. The Ni particles were evenly distributed on the alumina nanofibers, and the addition of silicon greatly enhanced the flexibility of the alumina nanofibers, which made them better catalyst support materials.

In addition, alumina nanofibers are also used in catalysis in many other ways. Moghadam et al. [111] prepared Ni/Al2O_3_-SiO_2_ catalysts with different SiO_2_/Al_2_O_3_ molar ratios by the sol–gel method. According to the BET, the specific surface area changed from 254 to 163.3 m^2^/g, and the grain size increased with the increase in the Si/Al molar ratio. In addition, the catalytic effect was the highest when the molar ratio of SiO_2_/Al_2_O_3_ was 0.5, and the conversion rate of CO_2_ was 82.38% at 350 °C. Jiao et al. [112] first prepared NbCl_5_-γ-Al_2_O_3_ nanofibers by an initial wet immersion method, with NbCl_5_ as the precursor of niobium. The results showed that the Nb_2_O_5_-γ-Al_2_O_3_ nanofibers could catalytically convert biomass to 5-carboxymethyl furfural with excellent catalytic performance. Ji et al. [113] prepared two catalysts, CoMo-I/γ-Al_2_O_3_ and CoMo-II/γ- Al_2_O_3_, using different active phase precursors. At the same time, Co and Mo single metal catalysts were selected to catalyze the decomposition of ammonia, and the catalyst support was γ- Al_2_O_3_. The results showed that the CoMo-I/γ- Al_2_O_3_ catalyst exhibited excellent catalytic activity compared with other catalysts.

### 4.4. Water Restoration

With large-scale urbanization and industrialization, water pollution has become one of the problems to be solved. The excellent properties of nanofibers give them great potential for the adsorption of dye pollutants. Qu et al. [114] elaborated on promising nanotechnology water treatment processes. The good surface area, photosensitivity, and catalytic and antibacterial activities of nanomaterials mean they are widely used in water treatment, such as water quality detection sensors, special adsorbents, solar disinfection, or decontamination films. Alumina ceramic nanofibers, as one type of ceramic nanofiber, also have great potential in removing different water pollutants. At the same time, alumina ceramic nanofibers exhibit morphological continuity, making them easy to separate from the treated water, avoiding more water contamination due to the process itself.

**Figure 12:**
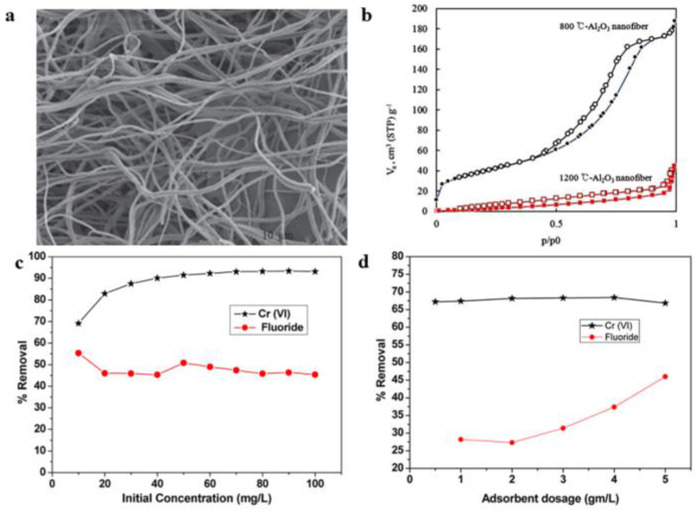
(**a**) SEM diagram of the alumina nanofibers. (**b**) N_2_ adsorption−desorption isotherm of the alumina nanofibers. (**c**) Effect of the metal ion concentration change on the chromium and fluorine adsorption effect. (**d**) Alumina nanofibers of the chromium and fluoride ion removal percentage figure curve along with the change in the dose. (**a**,**b**) Reprinted with permission from reference [115]. (**c**,**d**) Reprinted with permission from reference [116].

Researchers have prepared alumina nanofibers with different structures and compositions to adsorb dyes as well as some water contaminants. Kim et al. [115] obtained alumina nanofibers by sol–gel and electrospinning, as shown in Figure 12a,b. The characterization results showed that the method successfully prepared alumina nanofibers with diameters between 102 and 378 nm. In addition, the alumina nanofibers showed excellent adsorption properties for methyl orange dye. Mahapatra et al. [116] used electrospinning technology to produce alumina nanofibers, as shown in Figure 12c,d. Chromium (VI) and fluoride ions were removed from the aqueous system by using the prepared alumina nanofibers as an adsorbent. The study found that the maximum absorption of the above two ions of the electrospun alumina nanofibers was 6.8 and 1.2 mg/g, respectively. Mukhish et al. [117] prepared ultrafine alumina–silica nanofiber membranes by electrospinning. The membrane can be used to adsorb reactive red −120 dyes in the water system. The results show that the maximum capacity of the ultrafine alumina–silica nanofiber flexible self-supporting film was 884.95 mg/g. At the same time, the flexible membrane could be reused many times through the liquid phase separation.

### 4.5. Energy Storage Field

In addition to high-temperature filtration, catalysis, water remediation, and other fields, alumina nanofibers are also widely used in energy storage, such as lithium battery electrodes and membranes.

**Figure 13:**
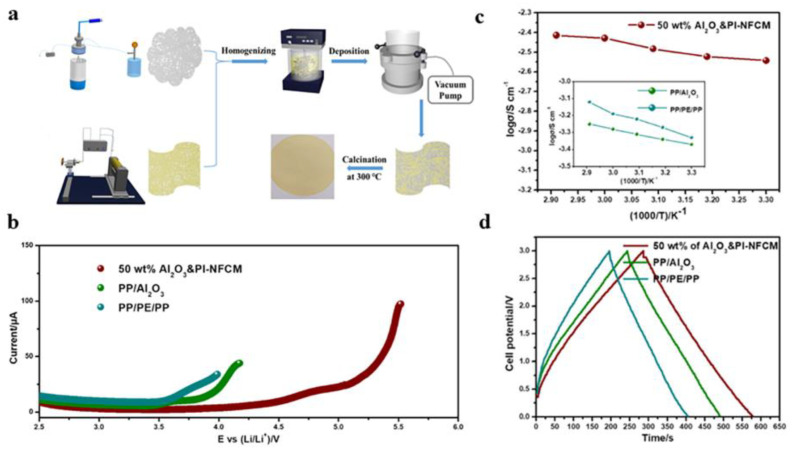
(**a**) Electrochemical performance diagram. (**b**) LSV curves of the batteries with different separators. (**c**) Ionic conductivity of the Al_2_O_3_ and PI NFCM−assembled batteries at different temperatures. (**d**) The GCD curve of different diaphragm assemblies of the LIC. (**a**–**d**) Reprinted with permission from reference [119].

Liu et al. [118] first spun alumina nanofibers and polyacrylonitrile nanofibers by electro blow spinning and then combined them by wet deposition to obtain a nanofiber composite membrane, which could be used as a lithium-ion battery separator. The experimental results showed that the composite battery separator had good thermal stability, and the battery safety was also improved. In addition, the study found that the introduction of alumina nanofibers made the composite battery separator exhibit better porosity, liquid electrolyte affinity, and lower interfacial resistance, thereby improving the battery cycle stability. Liu et al. (Figure 13) [119] prepared inorganic–organic nanofiber composite separators for lithium-ion capacitors (LICs) by wet, using electrospun alumina nanofibers and polyimide nanofibers(PI) as components. Alumina nanofibers provided rapid ion transfer properties and excellent fire resistance for this composite separator, and the presence of polyimide nanofibers enhanced the heat resistance and entanglement.

In addition to lithium-ion battery separators, alumina nanofibers can also be used in fuel cell composite proton exchange membranes. Borrell et al. [120] prepared alumina–carbon nanofiber nanocomposites using spark plasma sintering technology, which can be used in proton exchange membrane fuel cell bipolar plates. Compared with graphite bipolar plates, the mechanical strength of this dense ceramic–CNF nanocomposite material was three times higher, and the composite material reduced the thickness of the bipolar plate and thus the size of the fuel cell stack. Li et al. [121] used electrospinning technology and a high-temperature catalytic growth process to prepare alumina nanofibers using polytetrafluoroethylene as a catalyst. A new type of caterpillar-like alumina fiber and its composite proton exchange membrane (CAPEM) were obtained by doping the nanofibers into a non-fluorosulfonated aromatic polymer matrix by solution casting. The experimental results showed that the electrical conductivity of the CAPEM was as high as 0.263 S/cm at 80 °C and 100% relative humidity, and its thermal stability, water absorption, and swelling ratio were also improved.

Among the battery components, the electrode material is an important part. Since alumina nanofibers can interact with the electrode’s active ingredient, researchers have tried to add alumina nanofibers to the battery electrodes. Liu et al. [122] prepared Al_2_O_3_/C nanofibers by a one-pot electrospinning method. It could be used as an intermediate layer for lithium–sulfur batteries. Under the condition of 200 cycles, the battery still provided a 993 mAhg^−1^ discharge capacity, 73% of its initial capacity. Taleb et al. [123] designed and prepared graphene-coated alumina nanofibers, which can be used as electrochemically active materials. The initial discharge capacity and stability can be improved by combining the nanofiber with sulfur. Based on this property, the specific capacity of the composite material was increased and improved, up to ~900 mAh/g.

### 4.6. Other Applications

In addition to the above applications, alumina nanofibers have several other applications, such as sound absorption and as a substrate to grow a metal–organic framework (MOF). For example, Jia et al. [124] developed a super elastic SiO_2_-Al_2_O_3_ composite fiber sponge using solution blow spinning technology. The composite fiber sponge had excellent thermal insulation and sound absorption performance. Due to the low density and layered structure of the sponges, they had excellent sound absorption performance; the noise and sound waves were absorbed many times in the sponge. The sound absorption capacity and noise reduction coefficient were affected by the thickness of the sponge and were generally positively correlated. The increase in the sound absorption capacity and noise reduction coefficient greatly improved the sound absorption effect of the materials. Liang et al. [125] prepared flexible self-supported MOF fiber pads by water or the solvent–thermal reaction using electrospun alumina nanofibers as the matrix. The prepared MOF fiber pad had a large surface area and pore accessibility, which opens up new opportunities for the development of MOF materials.

Alumina nanofibers have some applications in the field of bioengineering. Dutta et al. [126] modified the AAO membrane based on a nanoporous anodized aluminum oxide (AAO) membrane using the biochemical characteristics of collagen nanofibers or the amino group provided by silanization of (3-amino-propyl) triethoxysilane (APTES) to obtain a permeable scaffold material that also promoted cell adhesion. The experimental results showed that the keratinocytes adhered well to the AAO membrane. When the AAO membrane was in contact with the E. coli suspension for 20 h, it was successful in preventing bacterial penetration. Ke et al. [127] first obtained Boehmite nanofibers by the steam-assisted wet gel conversion process, then coated the Boehmite nanofibers on the surface of the α-alumina carrier, and then converted the Boehmite nanofibers into γ-Al_2_O_3_ attached to the carrier surface by calcining the Boehmite nanofibers. Finally, silyl groups were introduced to modify the surface of the prepared alumina nanofiber membrane to obtain a high-efficiency protein separation membrane based on the ceramic nanofiber. The experimental results showed that the alumina nanofiber membrane could trap 100% of the BSA protein and 92% of the cellulase protein. At a concentration of 400 ppm, the nanofiber membrane also retained 75% of the trypsin protein and maintained the permeability of 48 L h^−1^ m^−2^ bar^−1^. Toloue et al. [128] added 1–5% wt to the PHB-CTS alloy solution by the electrospinning process. The composite scaffold of poly (3-hydroxybutyrate) chitosan/alumina nanowires was prepared by alumina nanowires. The results showed that the addition of alumina nanowires increased the crystallinity and tensile strength of the scaffold, and the hydrophilicity became excellent. The MTT assay showed that the absorption value of the MG-63 cells was higher when cultured on a scaffold containing alumina nanowires. Moreover, the alkaline phosphatase secretion of the composite scaffolds was higher than that of pure PHB scaffolds, which can be used as scaffolds for bone tissue engineering applications.

## 5. Conclusions and Outlook

In conclusion, alumina ceramic nanofibers have shown significant progress in their preparation methods and applications in various fields over the past decade. Electrospinning, solution blow spinning, and centrifugal spinning are among the main methods used for the fabrication of alumina ceramic nanofibers. The broad application prospects of alumina ceramic nanofibers highlight their potential as advanced functional materials with diverse applications. Continued research and development in this area are expected to further expand the range of applications for alumina ceramic nanofibers and contribute to the advancement of various technologies in fields such as energy, environment, and materials science.

However, while there has been significant progress in the preparation and applications of alumina ceramic nanofibers, there are also some development directions and opportunities that can be explored further. Here are some potential areas of focus.

1. Improved scalability and cost effectiveness: Although various methods such as electrospinning, solution blow spinning, and centrifugal spinning have been used for the fabrication of alumina ceramic nanofibers, there is still a need for scalable and cost-effective manufacturing processes. Developing methods that can produce alumina ceramic nanofibers in large quantities at a reasonable cost will enable their widespread commercial applications.
2. Enhanced properties and performance: Further research can focus on improving the properties and performance of alumina ceramic nanofibers. This can involve tuning the composition, morphology, and surface properties of the nanofibers to achieve the desired characteristics such as a higher thermal stability, mechanical strength, and electrical conductivity. This can open up new possibilities for advanced applications in areas such as energy storage, sensors, and electronics.
3. Novel applications: While alumina ceramic nanofibers have found applications in various fields such as heat insulation, filtration, catalysis, and energy storage, there may be other untapped areas where they can be utilized. Exploring novel applications of alumina ceramic nanofibers in emerging fields such as flexible electronics, aerospace, and environmental monitoring can provide new opportunities for their utilization.

In summary, there are several potential directions for further development and opportunities for alumina ceramic nanofibers. Continued research and innovation in these areas can lead to advancements in the field and enable new applications for these promising materials.

## Figures and Tables

**Table 1:** Preparation of the alumina spinning gel and the spinning conditions of the alumina nanofibers.

Aluminum Source	Polymers	Solvents	Additives	Aging Time	Calcination Conditions	References
AlCl_3_·6H_2_O Al(NO_3_)_3_·9H_2_O Al(O-i-Pr)_3_ Al	PEO	H_2_O	HNO_3_	/	After the temperature rose to 450 °C at 10 °C/min, the temperature was kept for 12 h and then for 0.5 h at 700 °C, 800 °C, and 900 °C, respectively.	4
AlCl_3_ Al	/	H_2_O	SiO_2_	10 h	After being kept at 200 °C for 4 h, the temperature was heated by 240 °C/h to 1200 °C for 4 h.	15
H_3_AlBO_2_	PVA	H_2_O	/	5 h	The temperature was raised from 1000 °C to 1200 °C by 240 °C/h, and the heat was kept for 2 h.	44
AlCl_3_	PVP	H_2_O C_2_H_5_OH	/	4 h	It was dried in a vacuum of 100 °C for 24 h and then in air at 450 °C, 900 °C, and 1100 °C for 5 h.	45
Al(NO_3_)_3_·9H_2_O AlCl_3_·6H_2_O Al(O-i-Pr)_3_ Al	PEO	H_2_O	HNO_3_	12 h	After drying for 24 h at 80 °C and calcination at different temperatures for 0.5 h, the heating rate was 10 °C min^−1^, except for 12 h at 450 °C.	48
Al_2_(SO_4_)_3_·18H_2_O C_6_H_9_AlO_6_	PVP	C_2_H_5_OH	(CH_3_COO)_2_Ba CH_3_COOH	/	The temperature was heated to 1000 °C at the rate of 5 °C min^−1^, and the heat was kept for 2 h.	49
AlCl_3_·6H_2_O Al(O-i-Pr)_3_	PVP	H_2_O	C_2_H_5_OH C_4_H_6_O_6_ HCl	/	It was dried at 70 °C for 48 h, then the temperature was raised by 2 °C/min to 600 °C for 1.5 h and then by 10 °C/min to 800 °C for 1.5 h.	50
AlCl_3_·6H_2_O Al(NO_3_)_3_·9H_2_O Al(O-i-Pr)_3_ Al	PVP	H_2_O	/	/	The temperature rose by 10 °C/min to 800 °C, and the heat was kept for 2 h.	51
Al(NO_3_)_3_·9H_2_O	PVP	H_2_O C_2_H_5_OH	/	1 h	It was heated up from 500 °C to 1200 °C and held for 2 h.	76
AlCl_3_·6H_2_O Al	PVA	H_2_O	mSiO_2_·nH_2_O	12 h	It was dried in air at 70 °C for 12 h; then, it was heated at a heating rate of 4 °C/min and calcined for 2 h.	77
(Al_2_(SO_4_)_3_·(14–18)H_2_O	/	H_2_O	NaOH	/	It was annealed in the air at 600 °C for 1 h or 1200 °C for 5 h.	85
Al	PEO PVP	H_2_O	HCOOH CH_3_COOH CuSO_4_	1 h	The temperature was raised by 1 °C/min to 600 °C, 700 °C, and 800 °C, and the heat was kept at each temperature for 2 h.	92
AlCl_3_	PVP	H_2_O	C_6_H_12_N_4_ (CH_3_COO_2_Zn DMF	/	The temperature was raised by 2.2 °C/min to 700 °C, with heat preservation for 2 h.	93
Al_2_O_3_	/	H_2_O	RhCl_3_·3H_2_O	/	It was dried overnight at 60 °C.	96
AlCl_3_·6H_2_O	PVP	H_2_O	CeCl_3_·7H_2_O C_2_H_5_OH	/	It was kept at 1000 °C for 4 h.	97
AlCl_3_	/	H_2_O	C_18_H_41_NO_7_S	1.5 h	The temperature was raised by 1.5 °C/min to 1500 °C, with heat preservation for 2 h.	100
α-Al_2_O_3_	PAA	H_2_O	PS 3Al_2_O_3_·2SiO_2_	1 h	The temperature was kept at 1550 °C for 2 h, and the residence time was 200 °C (2 h) and 500 °C (1 h).	101
Al_2_O_3_	Agarose solution	/	/	/	It was kept at 1575 °C for 4 h.	102
Aluminum salt Al(NO_3_)_3_·9H_2_O	/	H_2_O	C_8_H_21_NO	/	The temperature was raised by 1 °C/min to 500 °C, with heat preservation for 5 h.	107

## Data Availability

Not applicable.
